# Polysaccharides from* Trichosanthes Fructus* via Ultrasound-Assisted Enzymatic Extraction Using Response Surface Methodology

**DOI:** 10.1155/2017/6160785

**Published:** 2017-09-25

**Authors:** Fujia Chen, Dahong Li, Hongqi Shen, Chunhong Wang, Enzhong Li, Huihui Xing, Li Guo, Qingchun Zhao, Junhao Shi, Hoang Nguyen, Jiayang Liu

**Affiliations:** ^1^College of Biotechnology and Food Engineering, Huanghuai University, Zhumadian 463000, China; ^2^College of Chemistry and Pharmaceutical Engineering, Huanghuai University, Zhumadian 463000, China; ^3^College of Liberal Arts, Department of Biology, Mercer University, Macon, GA, USA

## Abstract

An efficient procedure for ultrasound-assisted enzymatic extraction of crude polysaccharides from* Trichosanthes Fructus *(crude TFP) using response surface methodology (RSM) was developed. The Box–Behnken design was applied to optimize the effects of pH (*X*_1_), enzyme amount (*X*_2_), extraction temperature (*X*_3_), and liquid-to-solid ratio (*X*_4_) on the extraction. The statistical analysis indicated that the independent variables (*X*_4_, *X*_2_, and *X*_3_), the quadratic coefficients (*X*_1_^2^, *X*_2_^2^, *X*_3_^2^, and *X*_4_^2^), and the interaction coefficient (*X*_1_*X*_3_) had significant impact on the yield of crude TFP. The optimal conditions were determined as follows: pH 4.5, enzyme amount 5000 u/g, extraction temperature 45°C, and liquid-to-solid ratio 30 ml/g. The experimental yield of crude TFP was 6.58%, which was very close to the predicted yield of 6.71%. TFPI was then purified and characterized with Sephadex G-100 column, UV-Vis, GPC, and FT-IR. The average molecular weight of TFPI was calculated to be 1.49 × 10^5^ Da. TFPI exhibited strong reducing power and possessed not only remarkable scavenging activities against ABTS^•+^ and DPPH radicals, but also high antitumor activities in C4-2, DU145, and PC3 cells. The results suggest that* Trichosanthes Fructus* and TFPI could be a novel potent natural medicine with antioxidant and antitumor activities.

## 1. Introduction

As the ripened fruit of* Trichosanthes kirilowii* Maxim, which is a perennial vine belonging to* Trichosanthes* genus of cucurbitaceous plants known as snake gourd fruit,* Trichosanthes Fructus* has been used in traditional Chinese medicine for the treatment of cerebrovascular and cardiovascular diseases due to its various pharmacological activities, such as dispelling phlegm to relieve cough and chest stuffiness [1]. In the past years, much attention has been paid to polysaccharides in* T. Fructus* (TFPs) as major compounds with potential biological activities. Their significant hypoglycemic, antioxidant, and immunoenhancing activities have been recently documented [2]. However, antitumor activities of these polysaccharides have yet to be explored.

Extraction method can significantly influence not only the content and yield of polysaccharides, but also their structural characteristics and bioactivities [3]. Ultrasonic extraction is an effective approach to disrupt cell walls, thereby improving the efficiency of mass transfer and penetration [4]. Enzymatic extraction, meanwhile, can attain better target compounds with decomposition reduced at a lower cost [5]. Consequently, ultrasound-assisted enzymatic extraction (UAEE) can be used to extract polysaccharides from* T. Fructus *by optimizing several independent variables and their interactions method via response surface methodology (RSM) [6].

We therefore studied the effect of ultrasound-assisted enzymatic extraction (UAEE) parameters on the yield of crude polysaccharide from* T. Fructus *(crude TFP) using Box–Behnken design (BBD) with 4 factors and 3 levels. For the first time, the structure and bioactivities of the purified polysaccharide from* T. Fructus *(e.g., TFPI) were investigated, and its antioxidant and antitumor activities were assessed* in vitro* as well. In this study, we observed that both crude TFP and TFPI showed considerable antitumor activity against 5 prostate cancer cell lines including LNCaP, 22RV1, C4-2, DU145, and PC3 cell, thus representing a novel kind of natural medicines for potential biological use.

## 2. Materials and Methods

### 2.1. Chemicals and Reagents

*T. Fructus* was purchased from a local traditional Chinese medicine market in Henan province, China. Sephadex G-100 column was obtained from GE Healthcare (Beijing, China). 3-(4,5-Dimethylthiazol-2-yl)-2,5-diphenyltetrazolium bromide (MTT), 2,2′-azinobis-(3-ethylbenzothiazoline-6-sulfonic acid) (ABTS), 1,1-diphenyl-2-picrylhydrazyl (DPPH), and ascorbic acid (V_C_), were obtained from Sigma (St. Louis, MO, USA). Cellulase (10,000 u/g) was purchased from Jinsui Biological Technology Co. Ltd. (Shanghai, China). All other analytical-grade chemicals were obtained from Nanjing Reagent Co. Ltd. (Nanjing, China).

### 2.2. Extraction of Crude TFP from* T. Fructus*

During the ultrasound-assisted enzymatic extraction (UAEE), the crude TFP was processed simultaneously by ultrasonic wave and enzymes. Firstly, the dried and milled* T. Fructus* were pretreated with acetone in a Soxhlet system for 24 h to remove the pigments and fats [7]. After being vacuum dried at 55°C, each pretreated sample (5.0 g) was put into a 500 ml flask and then extracted with cellulase at varying pH, ranging from 3.5 to 5.5, enzyme amount 1000–6000 u/g, extraction temperatures 35–65°C, and liquid-to-solid ratios 5–40 ml/g (v/w) in an ultrasonic cell disintegrator (JY92-2D, Ningbo Scientz Biological Technology Co., Ltd., Ningbo, China) for 30 min. After inactivating the cellulase in boiling water for 5 min, the extracted slurries were centrifuged for 15 min at room temperature and 4000 ×g (Allegra 64R, Beckman Coulter, Inc., Fullerton, USA). Then, the supernatants were collected, in which the yield of polysaccharides was roughly measured using the phenol-sulfuric acid method with D-glucose as a standard at 490 nm [8]. The crude TFP yield (%) was calculated as follows:

(1) $$\begin{array}{c} Yield\left(\;\%\;\right)=\frac{C\times V}{W}\times 100, \end{array}$$

where *C* (g/ml) is the concentration of polysaccharide solution, *V* (ml) is the volume of polysaccharide solution, and *W* (g) is the weight of dried sample.

### 2.3. Single Factor Experimental Design

The effects of pH, enzyme amount, extraction temperature, and liquid-to-solid ratio were first studied in an ultrasonic cell disintegrator for 30 min by a single factor design as follows: one experimental factor was changed while the other experimental factors were kept constant (Liu et al., 2015). The effect of each experimental factor was appraised by the yield of the crude TFP. All the experiments were repeated three times.

### 2.4. Box–Behnken Design and Statistical Analysis

On the basis of preliminary single factor experimental design, a Box–Behnken design (BBD) with four variables (*X*_1_, pH; *X*_2_, enzyme amount; *X*_3_, extraction temperature; *X*_4_, liquid-to-solid ratio) at three levels was used to further determine the optimal UAEE condition of crude TFP extraction [5, 9].  Table 1 showed that each independent variable was prescribed into three levels, coded +1 (high value), 0 (intermediate value), and −1 (low value), respectively. As shown in Table 2, the whole design consisted of 29 experimental points, and all the experiments were carried out at random to minimize systematic errors. All trials were carried out in triplicate. Data from BBD were analyzed by multiple regressions to fit the following quadratic polynomial model:

(2) $$\begin{array}{c} Y=\beta_{0}+\overset{4}{\underset{i=1}{\sum }}\beta_{i}X_{i}+\overset{4}{\underset{i=1}{\sum }}{\beta_{i}}_{i}{X_{i}}^{2}+\overset{4}{\underset{i<j=2}{\sum }}{\beta_{i}}_{j}X_{i}X_{j}, \end{array}$$

where *Y* is the predicted response, *X*_*i*_ and *X*_*j*_ are the coded independent variables, and *β*_*i*_, *β*_0_, *β*_*i*__*i*_, and *β*_*i*__*j*_ represent the coefficients of the linear, constant, quadratic, and interaction, respectively. *β*_*i*_ is the main effect. Design-Expert software (version 8.0.6.1, State-East, Inc., Minneapolis, USA) was utilized for the experimental design, data analysis, and model building. According to the analysis of variance (ANOVA), the effect and regression coefficients were measured, and three-dimensional (3D) surface and contour plots were generated.

### 2.5. Isolation and Preliminary Characterization of TFP I

The supernatant of crude TFP obtained under optimal condition was filtered and concentrated with a rotary evaporator (RE-5299, Yarong Technology and Science Inc., Shanghai, China) at 55°C under reduced pressure. Subsequently, the aqueous solution was deproteinized with Sevag reagent (chloroform : n-butyl alcohol = 4 : 1, v/v) for four times [10]. After removing the organic solvents, the solution was precipitated with ethanol up to 80% and incubated at 4°C overnight. The precipitate was collected by centrifugation and dialyzed (20,000 Da) against distilled water for 48 h successively. Then, the treated polysaccharide was lyophilized and purified by gel filtration chromatography (GFC) on a Sephadex G-100 column (80 cm × 1.6 cm i.d.). The column was eluted with distilled water at a flow rate of 0.5 ml/min, and 5 ml of each fraction was collected in each tube. The collected polysaccharides were monitored by phenol-sulfuric assay at 490 nm. The fraction I was freeze-dried and designated as TFPI for further structural characterization and bioactivity assay.

Absorption spectra in the range of 190–700 nm were recorded with a UV-Vis spectrophotometer (UV-2550, Shimadzu, Japan). Approximately 20 mg of TFPI was dried under an infrared lamp and then ground with KBr (1 : 100) and pressed into pellets prior to FT-IR analysis with Nicolet IS5 infrared spectrometer (Thermo Nicolet Co., USA) in the region of 4000–400 cm^−1^. Data were processed using Nicolet Omnic 8.0 software [11].

The molecular weight of TFPI was determined by high performance liquid gel permeation chromatography (HPGPC, Waters-1525, Waters, USA), which was equipped with a Waters-2410 refractive index detector (RID), and performed on two Ultrahydrogel™ linear columns (7.8 × 300 mm, Waters, USA) in serial. The injection volume was 30 *μ*l. Then, the columns were maintained at 30°C and eluted with 0.1 N sodium nitrate at the flow rate of 0.8 ml/min. Column calibration was performed with standard T-series dextrans (MW: 2700, 9700, 21,400, 36,800, 133,800, 401,000, and 2000,000 Da). The calibration curve of log⁡MW (logarithm of their respective molecular weight) of standard dextrans against their retention time (*T*) was obtained (log⁡MW = −0.526*T* + 13.7, *R*^2^ = 0.9981).

### 2.6. Antioxidant Activities* In Vitro*

The total reducing powers of the crude TFP and TFPI were determined based on a reported method [12]. The samples were dissolved in double distilled water to form sample solutions. Then, the solutions (1 ml) were thoroughly mixed with 2.5 ml phosphate buffer (0.2 M, pH 6.6) and 2.5 ml of 1% (w/v) potassium ferricyanide [K_3_Fe(CN)_6_], and the reaction mixture was reacted for 20 min at 50°C. Subsequently, 2.5 ml of 10% (w/v) trichloroacetic acid (TCA) solution was added to the mixture and centrifuged for 10 min at 3000 ×g. The supernatant (2.5 ml) was added to a test tube, followed by addition of 2.5 ml double distilled water and 0.5 ml of 0.1% (w/v) FeCl_3_. After 10 min of reaction, the absorbance at 700 nm was quantified with a spectrophotometer. The higher absorbance of the mixture reflects the greater reducing power. Ascorbic acid (Vc) was used as a positive control to compare the reducing power.

The ABTS^•+^ radical scavenging abilities of TFPs (including crude TFP and TFPI) were performed according to the method by Thambiraj et al. (2015) with slight modifications. The ABTS^•+^ reaction mixture was an aqueous solution consisting of ABTS^•+^ (7 mM) and potassium persulfate (2.45 mM), and the stock solution was incubated in the dark at room temperature for 14–16 h. The stock solution was diluted with PBS (pH = 7.4) to obtain the absorbance of 0.700 ± 0.004 measured at 734 nm before use. After the ABTS^•+^ solution (4.0 ml) was mixed with the tested sample solution (0.2 ml) for 20 min at room temperature, the absorbance was immediately analyzed at 734 nm. Vc served as a positive control. The ABTS^•+^ radical scavenging ability was expressed as follows:

(3) $$\begin{array}{c} \text{Scavenging ability}\left(\;\%\;\right)=\left[\;\frac{\left(A_{0}-A\right)}{A_{0}}\;\right]\times 100, \end{array}$$

where *A*_0_ is the absorbance of the blank control (without sample) and *A* is the absorbance of the sample.

The DPPH free radical scavenging ability was suitable for manifesting and evaluating the potential radical scavenging activities of antioxidants by colorimetry [13]. Briefly, the test solution of sample (1.0 ml) was mixed with 2.0 ml of DPPH solution (0.1 *μ*M in ethanol). Then, the mixture was shaken vigorously and incubated in darkness at room temperature for 30 min. The absorbance of the corresponding reaction solution was determined at 517 nm. Vc and double distilled water were employed as the positive and blank control, respectively. All the tests were performed in triplicate. The DPPH scavenging ability was calculated according to the following equation:

(4) $$\begin{array}{c} \text{Scavenging ability}\left(\;\%\;\right)=\left[\;\frac{\left(A_{0}-A\right)}{A_{0}}\;\right]\times 100, \end{array}$$

where *A*_0_ is the absorbance of the blank control and *A* is the absorbance of the sample.

### 2.7. Antitumor Activities of TFPs (Crude TFP and TFPI)* In Vitro*

LNCaP, 22RV1, C4-2, DU145, and PC3 human prostate cancer cell lines were purchased from ATCC company and were cultured in DMEM supplemented with 10% newborn bovine serum (NBS), 100 U/ml of penicillin, and 100 *μ*g/ml of streptomycin. All the cell lines were employed to evaluate the antitumor activities of TFPs (crude TFP and TFPI) by MTT assays [14]. Briefly, exponentially growing cells at a density of 1 × 10^5^ cells/ml were plated in 96-well plate and were inoculated at 37°C for 24 h to acquire adherent cells. Then, the cells were treated with TFPs solutions at different concentrations (2, 4, 8, and 16 *μ*M). The cells were then incubated for 24 h, 48 h, and 72 h, respectively. Then, 50 *μ*l of MTT solution (1.0 mg/ml in PBS) was added and the cells were incubated for 4 h at 37°C. After incubation, the supernatant was removed, and the formazan crystals were dissolved with DMSO (100 *μ*l). Absorbance at 490 nm was determined by a microplate reader (Thermo Multiskan EX, USA). Cisplatin was used as the positive control at a final concentration of 5 *μ*g/ml. The inhibition rate was calculated as follows:

(5) $$\begin{array}{c} \text{Inhibition rate}\left(\;\%\;\right)=\left(\;\frac{1-A}{A_{0}}\;\right)\times 100, \end{array}$$

where *A*_0_ and *A* are the absorbance of untreated cells and treated cells, respectively.

### 2.8. Statistical Analysis

All data are presented as means ± standard deviation (SD). The antitumor experiments were repeated five times, and the other experiments were repeated three times. Statistical analysis was carried out by ANOVA. Difference was considered to be statistically significant if *p* < 0.05.

## 3. Results and Discussion

### 3.1. Single Factor Experimental Design

**Figure 1:**
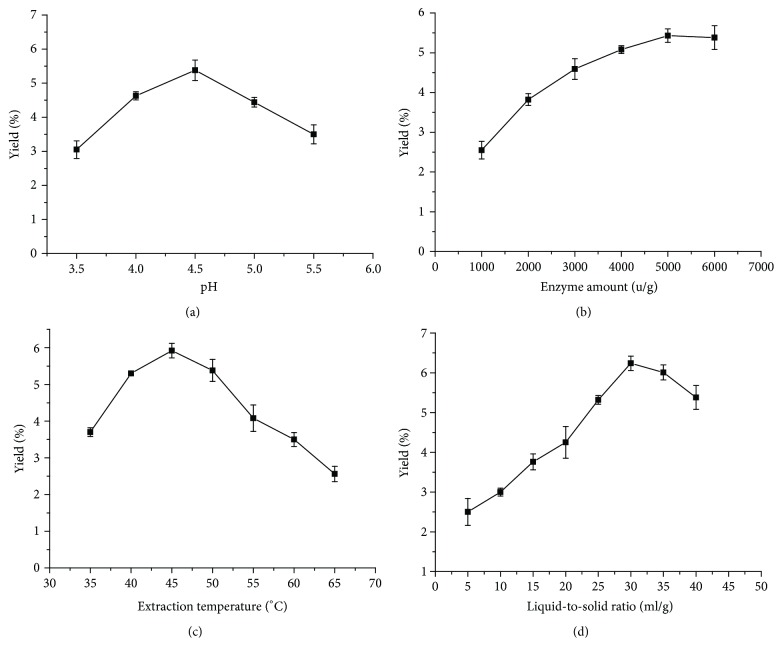
Effect of different pH (a), enzyme amount (b), extraction temperature (c), and liquid-to-solid ratio (d) on extraction yield of crude TFP. (a) Enzyme amount, extraction temperature, liquid-to-solid ratio, and extraction time were constant at 6000 u/g, 50°C, 40 ml/g, and 30 min, respectively. (b) pH, extraction temperature, liquid-to-solid ratio, and extraction time were constant at 4.5, 50°C, 40 ml/g, and 30 min, respectively. (c) pH, enzyme amount, liquid-to-solid ratio, and extraction time were constant at 4.5, 6000 u/g, 40 ml/g, and 30 min, respectively. (d) pH, enzyme amount, extraction temperature, and extraction time were constant at 4.5, 6000 u/g, 50°C, and 30 min. Error bars represent standard deviation of the means (*n* = 3).

Effects of single factor on extraction yield of crude TFP was depicted in Figure 1. Despite the fact that the ultrasound extraction would generate a lot of heat under the process, the temperature was kept to a given temperature by shorter time (30 min) and intermittent sonicated [15]. To investigate the effect of different pH conditions on the yield of crude TFP, the extraction process of UAEE was performed for 30 min at different pH conditions with the enzyme amount 6000 u/g, extraction temperature 50°C, and liquid-to-solid ratio 40 ml/g (Figure 1(a)). The extraction yield of crude TFP increased with pH increasing from 3.5 to 5.5 with a peak value of 5.38% at pH 4.5. However, further increase of pH led to a decrease in the yield of crude TFP. The pH of solutions may have great influence on the activities of different enzymes and their conformations [16]. The decrease in the yield of crude TFP might be ascribed to poor enzyme activities at the unsuitable pH value. Therefore, pH 4.5 was chosen for further experiments.

The amount of cellulase can also play an important role in the extraction of crude TFP [9]. As shown in Figure 1(b), the initial increase of cellulase from 1000 u/g to 6000 u/g resulted in an obvious increase in crude TFP yield with maximum value of 5.43% at enzyme dose of 5000 u/g. Taking crude TFP extraction yield and enzyme consumption into account [3], cellulase of 5000 u/g was believed to be enough for the extraction. By comparison, the highest crude TFP yield (5.43%) under optimum enzyme dose was found slightly lower than those under other conditions in Figures 1(a), 1(c), and 1(d), which might be caused by the impact of cellulase enzyme and the varied extraction conditions.

The procedure of UAEE was then performed for 30 min at different temperatures (35, 40, 45, 50, 55, 60, and 65°C) with the following condition: pH 4.5, enzyme amount 6000 u/g, and liquid-to-solid ratio 40 ml/g.  Figure 1(c) revealed that the extraction yield rose from 2.56% to 5.92% with the extraction temperature increasing from 35 to 45°C and peaked at 45°C. Further increase of temperature did not enhance the crude TFP yield, which was supported by the previous reports [9]. Apart from offering high diffusion coefficient and good solubility of polysaccharides in the solvent, the high extraction temperature can cause great enzyme activity loss as well [5]. Thus, 45°C was the most suitable extraction temperature in the present study.

Screening of the appropriate ratio of liquid-to-solid is very important during UAEE [6], which can facilitate the combination of the plant cells to the active site of the cellulase and the dissolubility of crude TFP in solvent. The effect of different liquid-to-solid ratios on the yield of crude TFP is shown in Figure 1(d), under the conditions of pH 4.5, enzyme amount 6000 u/g, extraction temperature 50°C, and extraction time 30 min. With an increasing ratio from 5 ml/g to 40 ml/g, the yield of crude TFP increased markedly, reaching the maximum value of 6.24% at 30 ml/g. Therefore, 30 ml/g was selected as the optimal ratio of liquid-to-solid for the following work.

### 3.2. Optimization of the Crude TFP Extraction Using RSM

In order to further identify the major variables influencing the TFP yield, response surface methodology (RSM) was then used to optimize the extraction process. The yield and activity of crude TFP are highly dependent on extraction conditions, whereupon the effects of four independent variables (*X*_1_, pH; *X*_2_, enzyme amount; *X*_3_, extraction temperature; *X*_4_, liquid-to-solid ratio) on crude TFP yield were further studied by response surface methodology (RSM) based on Box–Behnken design (BBD). All 29 experimental combinations and the response values (crude TFP yields) in the BBD are shown in Table 2, and runs 25–29 as five central replicates in the design were estimated by a pure error sum of squares. Using multiple regression analysis on the experimental data, the predicted response on crude TFP yield (*Y*) and the test variables were related by the following quadratic polynomial model according to actual value:

(6) Y=−181.32967+48.97033X1+4.18917×10−3X2+2.18920X3+1.01937X4−3.65×10−4X1X2−0.089X1X3−0.036X1X4+2.4×10−5X2X3−6.75×10−6X2X4−8.0×10−4X3X4−4.64967X12−3.19917×10−7X22−0.020547X32−0.012162X42.

The fitted regression equation was expressed in terms of surface and contour plots to visualize the relationships between the responses and the experimental levels of each factor and deduce optimal extraction conditions. Statistical analysis of each coefficient was checked by *F*-test and *p* value, and the analysis of variance (ANOVA) for the response surface model was shown in Table 3. The *F*-value (*F* = 35.72) and *p* value (*p* < 0.01) showed that the model was significant. The significance of the model was also determined by the test of lack of fit, while the *F*-value of 5.55 and the associated *p* value of 0.0566 were insignificant, respectively, indicating that the model was accurate enough to predict the relevant response [9, 17].

The goodness-of-fit of the model was also evaluated by the determination coefficient (*R*^2^ = 0.9728) and adjusted determination coefficient (Adj-*R*^2^ = 0.9455), which indicated that 97.28% of the variations could be illustrated by the fitted model and 94.55% of the total variations were explained by the model [17]. Furthermore, Adj-*R*^2^ was slightly smaller than *R*^2^ and the predicted determination coefficient (Pred-*R*^2^ = 0.8508) was also found to be smaller and very close to Adj-*R*^2^. This indicated that the observed data had a good correlation with that of prediction generated by the model [5, 9]. A fairly low coefficient variation value (CV%) represents the dispersion degree between predicted and observed values. In this study, the low CV% (3.93) clearly revealed a higher degree of precision and a better reliability of the observed values (Wu et al., 2013).

It could be seen from Table 3 that the linear coefficients *X*_4_ and *X*_2_ and the quadratic coefficient (*X*_1_^2^, *X*_2_^2^, *X*_3_^2^, and *X*_4_^2^) were very significant (*p* < 0.01), while the linear coefficient *X*_3_ and the interaction coefficient *X*_1_*X*_3_ had remarkable effects (*p* < 0.05). The other coefficients were not significant (*p* > 0.05). Consequently, the liquid-to-solid ratio (*X*_4_) was the major factor affecting the yield of crude TFP, followed by the enzyme amount (*X*_2_), extraction temperature (*X*_3_), and pH value (*X*_1_). All these statistical data above demonstrated that the model was reliable, precise, and adequate for prediction within the range of these variables.

**Figure 2:**
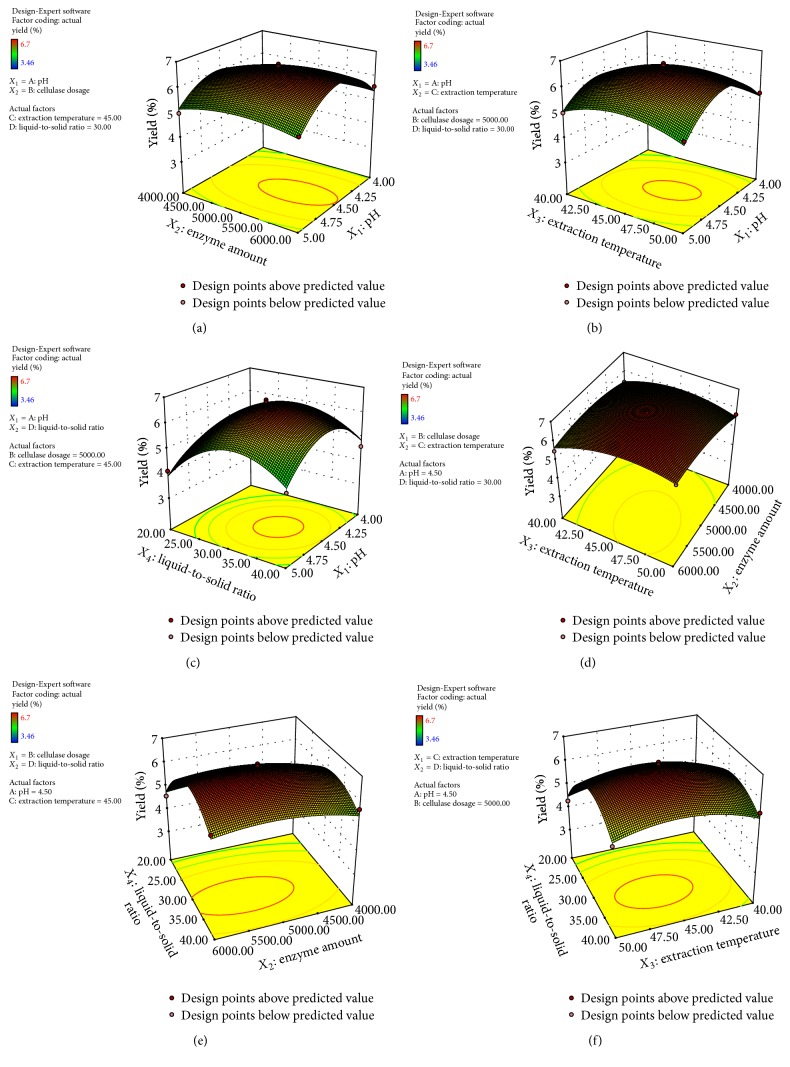
Response surface (3D) showing the effect of pH (*X*_1_), enzyme amount (*X*_2_), extraction temperature (*X*_3_), and liquid-to-solid ratio (*X*_4_) on extraction yield of crude TFP. (a) Interaction between pH and enzyme amount; (b) interaction between pH and extraction temperature; (c) interaction between pH and liquid-to-solid ratio; (d) interaction between enzyme amount and extraction temperature; (e) interaction between enzyme amount and liquid-to-solid ratio; (f) interaction between extraction temperature and liquid-to-solid ratio.

The interactions between the variables and the relationship between responses and experiment levels of each variable were depicted and visualized by three-dimensional (3D) response surface plot (Figure 2). The two tested variables were illustrated in one 3D surface plot while the others remained at 0 level listed in Table 1. As shown in Figure 2, the optimal extraction conditions for crude TFP extraction were predicted as follows: pH 4.49, enzyme amount 5373.44 u/g, extraction temperature 46.06°C, and liquid-to-solid ratio 32.25 ml/g. Under optimal conditions, the maximum predicted yield was 6.71%, which was very close to the experimental yield 6.58%, suggesting that the employed model was suitable for optimizing the crude TFP extraction conditions. For practical applications, however, extraction condition of pH value 4.5, enzyme amount 5000 u/g, extraction temperature 45°C, and liquid-to-solid ratio 30 ml/g might be recommended.

In this study, TFP extraction yield was determined with the phenol-sulfuric method because it has been extensively adopted in polysaccharide content determination due to its advantages, for example, easy operating and high sensitivity [8]. However, it should be noted that the obtained TFP yields might be overestimating the theoretical values of TFP by this method since it could detect monosaccharides and oligosaccharides in the samples as well. Furthermore, the ratio of total carbohydrates/reductive carbohydrates in the TFP samples should be clarified in the further study.

### 3.3. Purification and Characteristics of TFPI

**Figure 3:**
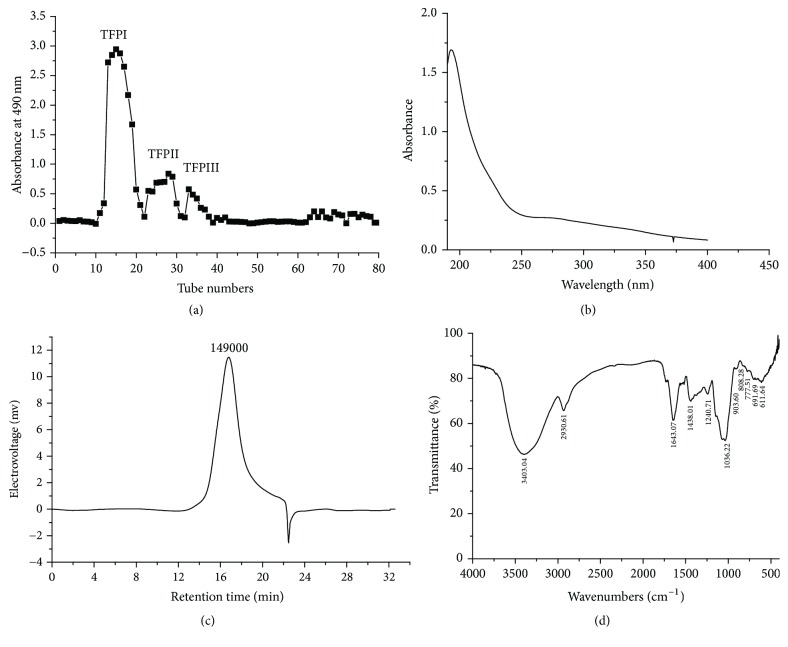
(a) Elution curves of crude TFP on a Sephadex G-100 column, three fractions were named as TFPI, TFPII, and TFPIII, respectively. (b) UV absorption spectrogram of TFPI. (c) HPGPC chromatogram of TFPI. (d) FT-IR spectra of TFPI.

Crude TFP was then isolated and purified by Sephadex G-100 column chromatography (Figure 3(a)), where three elution peaks TFPI, TFPII, and TFPIII were obtained, respectively. TFPI was found to be the most predominant among three elutions according to their peak shapes and thus collected for further structural analysis and antioxidant and antitumor experiments. No absorption peak was observed between 260 and 280 nm in the UV spectrum of TFPI (Figure 3(b)), indicating that TFPI did not contain proteins or nucleic acids [2]. The result of HPGPC in Figure 3(c) showed that the elution peak of TFPI was a single symmetric peak with a relatively narrow distribution, suggesting that TFPI was of chromatographic grade and identified to be a homogeneous polysaccharide. The negative peak might be solvent peak [18]. Molecular weight (MW) of TFPI was calculated to be 1.49 × 10^5^ Da according to the equation of the standard curve (log⁡MW = −0.526*T* + 13.7, *R*^2^ = 0.9981, *T* represented elution time) and retention time (16.21 min) of the elution peak. Standard dextrans were used as molecular markers.

The FT-IR spectra of TFPI (Figure 3(d)) revealed a wide and strong absorption peak at 3403 cm^−1^ corresponding to the O-H stretching vibration [2]. The weaker absorption peak at 2931 cm^−1^ was mainly contributed by the C-H (-CH_2_-, -CH_3_, and -CHOH) stretching vibration [2]. The absorption peak at 1643 cm^−1^ was caused by the bending mode of bound water [19], and the absorption peak at 1438 cm^−1^ was C-O stretching vibration [20]. The peak at around 1241 cm^−1^ was a symmetrical carbonyl stretching. The absorption peak at 1036 cm^−1^ was the characteristic peak of pyranoside and the weak small peak at 904 cm^−1^ indicated *β*-glucosidic bonds in TFPI [21]. All the structural characteristics of TFPI were quite different from the structure of TPP-1 previously reported by Chen et al. [2]. Such a result might be due to the different sources of* T. kirilowii* Maxim and also the different extraction methods.

### 3.4. Antioxidant Activity of TFPs* In Vitro*

**Figure 4:**
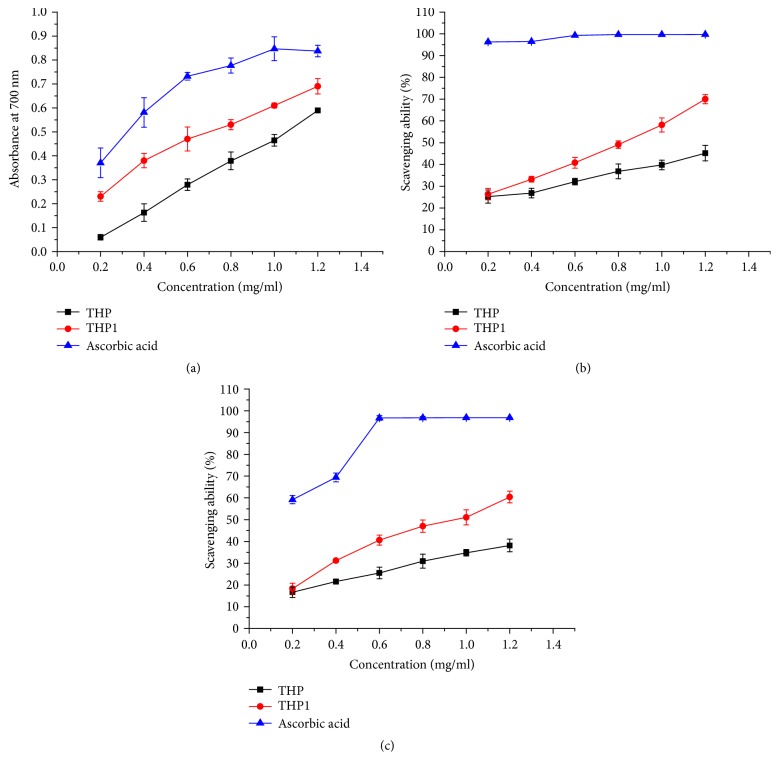
The antioxidant activities of crude TFP and TFPI* in vitro*. (a) The total reducing power. (b) ABTS radical scavenging activity. (c) DPPH free radical scavenging activity.

The total reducing power of natural compounds serves as a significant indicator of its potential antioxidant activity [13]. The presence of reducing agent such as polysaccharides would result in the transformation from Fe^3+^ to Fe^2+^ by donating an electron, reducing them into more stable and unreactive species [22]. Meanwhile, the reaction mixture turned yellow to blue as a result of the formation of Perl's Prussian blue, which can be monitored at 700 nm [23].  Figure 4(a) shows the reducing power of crude TFP, TFPI, and ascorbic acid (Vc, positive control) of varied concentrations. Ascorbic acid, the most effective and least toxic antioxidant, is involved in vital biological activities to reduce risk of cancer and cardiovascular diseases [24]. When the sample concentration reached 1.2 mg/ml, both crude TFP and TFPI exhibited obvious reducing power with the activities of 0.59 and 0.69, respectively, compared with that of ascorbic acid (0.84). It has been reported that the reducing properties are generally associated with the capacities of reacting with some precursors of peroxides to prevent their formation [25]. Based on that theory, TFPI could have a strong ability to donate electrons and reduce peroxide. Moreover, the reducing powers of all the two tested samples and positive control were closely correlated with their scavenging activities, which was consistent with previous research [26].

ABTS^•+^ radical, as a nitrogen center, has been widely used to test the antioxidant activity of natural extracts by monitoring the decreased green color caused by antioxidants [27].  Figure 4(b) described the scavenging effects of polysaccharides from* T. Fructus *and Vc as positive control on ABTS^•+^ radicals, which showed a concentration-dependent manner ranging from 0.2 to 1.2 mg/ml. The ABTS^•+^ radical scavenging activities were 45.2% and 70% for crude TFP and TFPI and 99.76% for Vc at concentration of 1.2 mg/ml, respectively.

The scavenging activity of TFP was further assessed with the dark purple DPPH with a maximum absorbance at 517 nm [17]. As shown in Figure 4(c), a similar trend was found for crude TFP, TFPI, and Vc as positive control, and the scavenging effects were increased in a concentration-dependent manner. At a concentration of 1.2 mg/ml, the DPPH free radical scavenging activities of crude TFP and TFPI were 38.14% and 60.40%, whereas that of Vc was as high as 96.87%. The DPPH scavenging activities were slightly lower than the ABTS^•+^ scavenging activities, which has also been observed in a previous report [28]. Polysaccharides are known to possess reducing properties, so they can also reduce ABTS and DPPH radicals; they have -OH groups and act as antioxidants [29]. Many human diseases are known to be caused by free radicals and the natural antioxidants can act as free radical scavengers [30].

### 3.5. Antitumor Activities of TFPI* In Vitro*

**Figure 5:**
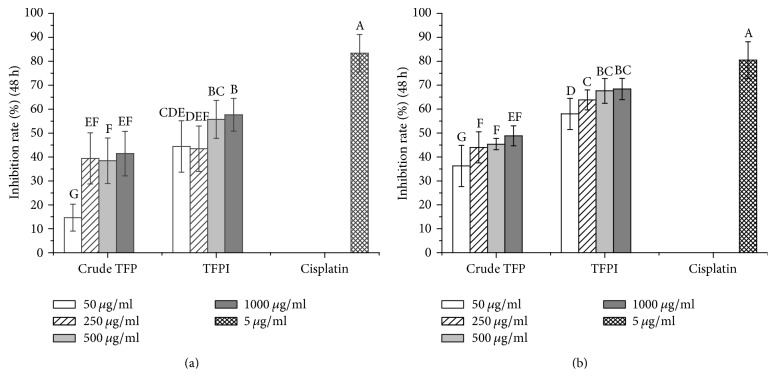
Inhibitory effects of crude TFP and TFPI* in vitro* against human prostate cancer LNCaP (a) and PC3 (b) cells at 48 h treatment. Data were means ± SD (*n* = 5) by one-way ANOVA. Different letters donated significantly different according to Duncan's test (*p* ≤ 0.05).

A number of polysaccharides have been reported with significant antitumor activity, but description on the antitumor activity of TFPs (both crude TFP and purified TFP) remains very little. Herein MTT assay was adopted for the antitumor activities of crude TFP and TFPI and the results are displayed in Figure 5. With cisplatin as the positive control, both crude TFP and TFPI exerted concentration-dependent inhibition activities on LNCaP and PC3 cells at 48 h. Compared with cisplatin (83.40% at 5 *μ*g/ml), the inhibition rates of crude TFP and TFPI at a high concentration of 1000 *μ*g/ml for LNCaP cells were 41.40% and 58%, while those for PC3 cells were 49% and 68%, compared with cisplatin (80.50% at 5 *μ*g/ml), respectively. Clearly, TFPI possessed relative higher antitumor activities* in vitro* than crude TFP, and the inhibition effects on PC3 cells were higher than those on LNCaP cells.

**Figure 6:**
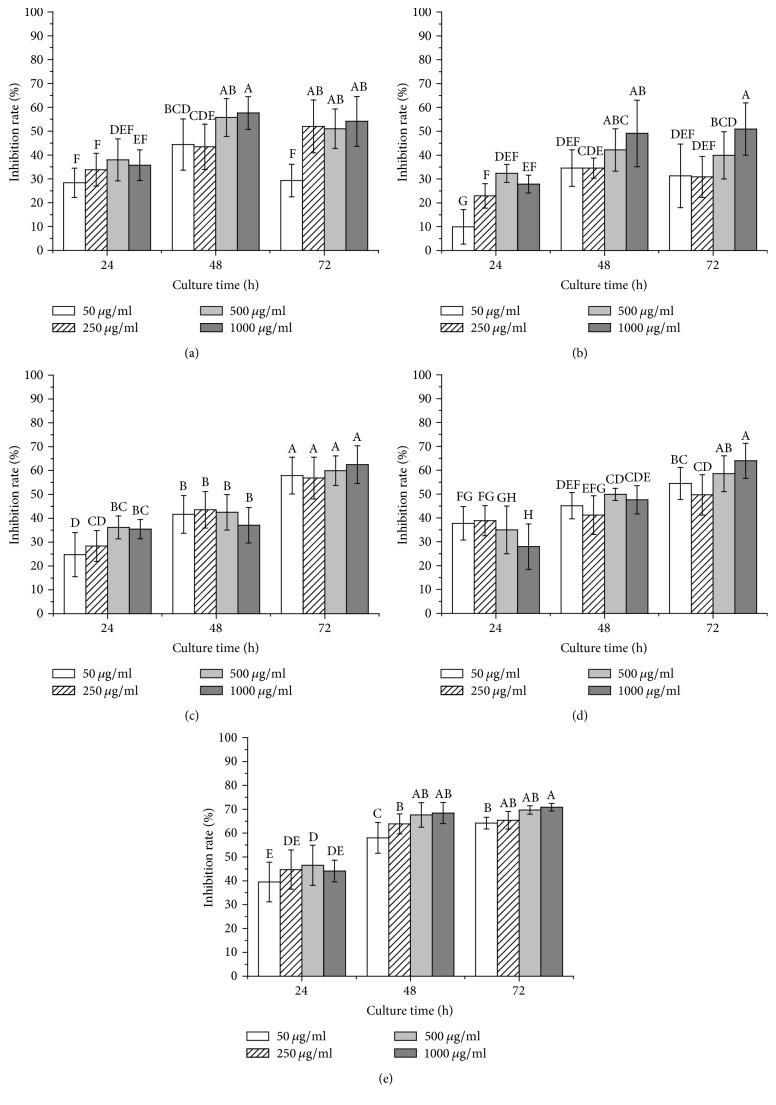
Antitumor bioactivity of TFPI against human prostate cancer LNCaP (a), 22RV1 (b), C4-2 (c), DU145 (d), and PC3 (e) cells cultured for 24 h, 48 h, and 72 h. Data were means ± SD (*n* = 5) by one-way ANOVA. Different letters were significantly different according to Duncan's test (*p* ≤ 0.05).

As shown in Figure 6, TFPI exhibited concentration and time-dependent inhibition activities* in vitro* against the five different human prostate cancer cells, including LNCaP, 22RV1, C4-2, DU145, and PC3 cells.

The inhibition rates of all the five cell lines were increased with prolonged culturing time during incubation. Particularly, compared with cultured for 24 h and 48 h, the inhibition rates were significantly increased in C4-2, DU145, and PC3 cells for 72 h. After 72 h incubation, the inhibition rates of TFPI for LNCaP, 22RV1, C4-2, DU145, and PC3 cells at a concentration of 1000 *μ*g/ml were 54.14%, 50.95%, 62.52%, 63.95%, and 70.90%, respectively. As one of the common cancers in man, prostate cancer is a threat to human health. Despite recent progress in diagnostic and multimodal therapies of initial prostate cancer, it is difficult to control the androgen-independent cases, such as DU145, PC3, and other advanced prostate cancers, which might cause high mortalities. Cisplatin is one of the most potent anticancer agents in chemotherapy associated with serious side effects, such as skin allergies and neuronal and kidney toxicity, because it interferes with DNA replication [31]. Therefore, it is very significant to seek the new antitumor lead compounds such as polysaccharides [32] from traditional medicinal plants to alleviate side effects of the chemotherapy. In this study, TFPI had a relatively stronger antitumor activity, which was far from the activities of the conventional pharmaceutical drugs cisplatin. So, the natural polysaccharides need structure modification to improve the efficacy in future work, as the obvious flaws of these polysaccharides, such as the structural heterogeneity, relatively lower active group, and poor solubility [33]. The antitumor activities of the polysaccharides may result from their abilities to modulate signaling pathways via regulating host's immune system or direct cytotoxic effects, such as inducing apoptosis of cancer cell, the effects of antiangiogenesis, and cell cycle arrest [34]. The polysaccharides are also capable of generating free radicals and oxidative stress, which are related to the antitumor activities of the polysaccharides [34, 35]. Therefore, polysaccharides from* T. Fructus* can be explored as a kind of natural medicine for potential antitumor treatment. The antitumor mechanisms of the polysaccharides have yet to be investigated in the future.

## 4. Conclusions

In summary, an efficient ultrasound-assisted enzymatic extraction (UAEE) was employed to extract polysaccharides from* Trichosanthes Fructus* by an optimized method using RSM for the first time. Results showed that pH, enzyme amount, extraction temperature, and liquid-to-solid ratio significantly affected the yield of crude TFP. The optimal extraction conditions with the highest yield (6.58%) were determined as follows: pH value of 4.5, enzyme amount of 5000 u/g, extraction temperature of 45°C, and liquid-to-solid ratio of 30 ml/g. The predominant fraction TFPI was isolated from crude TFP by Sephadex G-100 column chromatography with average molecular weight of 1.49 × 10^5^ Da and showed a strong reducing power of scavenging activities against ABTS^•+^ and DPPH radicals. Furthermore, relative higher antitumor activities in C4-2, DU145, and PC3 cells were confirmed for TFPI. Hence, it can be concluded that TFPI should be a kind of potent natural medicines with antioxidant and antitumor activities. Further works on the precise structures, functions, and mechanisms of TFPI are in progress.

## Figures and Tables

**Table 1:** Independent variables and experimental design levels in the Box–Behnken design for determining the optimal UAEE condition of crude TFP extraction.

Independent variables	Coded symbols	Levels		
		−1	0	1
pH	*X*_1_	4	4.5	5
Enzyme amount (u/g)	*X*_2_	4000	5000	6000
Extraction temperature (°C)	*X*_3_	40	45	50
Liquid-to-solid ratio (ml/g)	*X*_4_	20	30	40

**Table 2:** The BBD matrix with four variables and response values for the yield of crude TFP.

Run	*X*_1_ (pH)	*X*_2_ (enzyme amount, u/g)	*X*_3_ (extraction temperature, °C)	*X*_4_ (liquid-to-solid ratio, ml/g)	*Y* (yield of crude TFP, %)
1	4	4000	45	30	4.51
2	5	4000	45	30	4.99
3	4	6000	45	30	5.58
4	5	6000	45	30	5.33
5	4.5	5000	40	20	4.2
6	4.5	5000	50	20	4.29
7	4.5	5000	40	40	5.5
8	4.5	5000	50	40	5.43
9	4	5000	45	20	3.46
10	5	5000	45	20	4.11
11	4	5000	45	40	4.6
12	5	5000	45	40	4.53
13	4.5	4000	40	30	5.44
14	4.5	6000	40	30	5.5
15	4.5	4000	50	30	5.7
16	4.5	6000	50	30	6.24
17	4	5000	40	30	4.32
18	5	5000	40	30	5
19	4	5000	50	30	5.32
20	5	5000	50	30	5.11
21	4.5	4000	45	20	4.1
22	4.5	6000	45	20	4.58
23	4.5	4000	45	40	5.71
24	4.5	6000	45	40	5.92
25	4.5	5000	45	30	6.7
26	4.5	5000	45	30	6.56
27	4.5	5000	45	30	6.43
28	4.5	5000	45	30	6.63
29	4.5	5000	45	30	6.6
Optimal conditions	4.49	5373.44	46.06	32.25	6.71

**Table 3:** ANOVA of regression model for the yield of crude TFP.

Source	Coefficient estimate	Sum of squares	Degree of freedom	Standard error	Mean square	*F*-value	*p* value
Model	6.58	21.30	14	0.092	1.52	35.72	<0.0001^*∗∗*^
*X*_1_	0.11	0.14	1	0.060	0.14	3.21	0.0950
*X*_2_	0.22	0.61	1	0.060	0.61	14.26	0.0020^*∗∗*^
*X*_3_	0.18	0.38	1	0.060	0.38	8.88	0.0100^*∗*^
*X*_4_	0.58	4.03	1	0.060	4.03	94.49	<0.0001^*∗∗*^
*X*_1_ *X*_2_	−0.18	0.13	1	0.10	0.13	3.13	0.0988
*X*_1_ *X*_3_	−0.22	0.20	1	0.10	0.20	4.65	0.0490^*∗*^
*X*_1_ *X*_4_	−0.18	0.13	1	0.10	0.13	3.04	0.1030
*X*_2_ *X*_3_	0.12	0.058	1	0.10	0.058	1.35	0.2643
*X*_2_ *X*_4_	−0.068	0.018	1	0.10	0.018	0.43	0.5236
*X*_3_ *X*_4_	−0.040	0.006	1	0.10	0.006	0.15	0.7041
*X*_1_^2^	−1.16	8.76	1	0.081	8.76	205.76	<0.0001^*∗∗*^
*X*_2_^2^	−0.32	0.66	1	0.081	0.66	15.58	0.0015^*∗∗*^
*X*_3_^2^	−0.51	1.71	1	0.081	1.71	40.18	<0.0001^*∗∗*^
*X*_4_^2^	−1.22	9.59	1	0.081	9.59	225.22	<0.0001^*∗∗*^
Residual		0.60	14		0.043		
Lack of fit		0.56	10		0.056	5.55	0.0566
Pure error		0.040	4		0.010		
Cor total		21.90	28				
*R* ^2^		0.9728		Adep. precision		21.857	
Adj-*R*^2^		0.9455		CV%		3.93	
Pred-*R*^2^		0.8508		*r*		0.9863	

^*∗*^0.01 ≤ *p* < 0.05; ^*∗∗*^*p* < 0.01.
